# The Battle Against Pertussis: Discovery of Endogenous Human Proteins and Peptides as Toxin-Inhibitors

**DOI:** 10.3390/toxins18050208

**Published:** 2026-04-29

**Authors:** Stefanie Lietz, Holger Barth

**Affiliations:** Institute of Experimental and Clinical Pharmacology, Toxicology and Pharmacology of Natural Products, Ulm University Medical Center, 89081 Ulm, Germany

**Keywords:** *Bordetella pertussis*, pertussis toxin, AB-type protein toxin, toxin inhibitor, endogenous proteins, endogenous peptides, antimicrobial peptides, α_1_-antitrypsin, human defensins

## Abstract

The life-threatening disease pertussis, also known as whooping cough, is caused by a complex interplay of several virulence factors produced by the bacterium *Bordetella* (*B.*) *pertussis*. These include the AB-type protein toxin pertussis toxin (PT), the main causative agent of pertussis. After infection with *B. pertussis*, PT is released and binds to its human target cells, which internalize PT. The enzyme subunit of PT is then taken up into the cytosol, where it catalyzes the ADP-ribosylation of the α-subunit of inhibitory GTP-binding proteins from the Gαi type. This ultimately leads to the development of the characteristic clinical symptoms associated with pertussis. Pertussis is a vaccine-preventable but highly infectious respiratory disease, and especially younger children are prone to develop severe pertussis. Despite the vaccination, over the past few years, increasing case numbers have been reported globally. Moreover, treatment options are strongly limited to antibiotics and symptomatic treatment. Therefore, novel therapies against toxin-mediated diseases are urgently required, while AB-type toxins such as PT are promising pharmacological targets to combat these associated diseases. To identify novel pharmacological inhibitors for AB-type toxins, huge potential lies within the human proteome/peptidome. Endogenous protein or peptide inhibitors for bacterial toxins might have evolved as part of the innate immunity and are awaited to be discovered. The scientific community is committed to identify potential candidates through targeted screening or explorative hypothesis-driven approaches. This review summarizes the recent efforts in the identification and characterization of the human body’s own proteins and peptides that inhibit PT. PT-inhibiting peptides were found by unbiased screening of peptide libraries from human hemofiltrate or hypothesis-driven evaluation, and PT-neutralizing mechanisms were discovered in cell-based approaches. The identification of endogenous peptides and proteins, e.g., defensins and α_1_-antitrypsin, as potent inhibitors of PT paves the way towards the development of novel therapeutic options against pertussis.

## 1. Introduction

Over the past few years, increasing case numbers for several highly infectious diseases have been reported. In particular, bacterial infections such as pertussis or diphtheria are on the rise. Despite high vaccination coverage, several outbreaks were reported by the European Center for Disease Prevention and Control (ECDC), also in Western countries [1,2].

**Figure 1:**
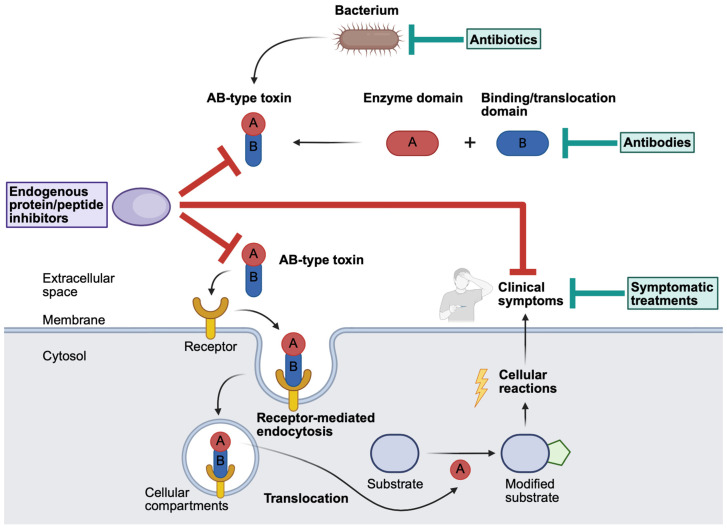
Structure and uptake of AB-type toxins and targets of conventional and novel therapies to combat toxin-mediated diseases. Bacteria produce AB-type protein toxins that are virulence factors and the causative agents of the symptoms after bacterial infection. AB-type toxins are built of two domains, an enzyme A-domain and a binding/translocation B-domain. After binding of the toxin via its B-domain to cell surface receptors, receptor-mediated endocytosis takes place, and the toxin ends up in cellular compartments. From those, the A-domain translocates into the cytosol and modifies its corresponding substrate, which causes specific cellular reactions and ultimately, clinical symptoms. Conventional therapies used to combat toxin-mediated diseases comprise the application of antibiotics and antibodies. Aside, symptoms are treated. Novel strategies comprise toxin-targeted approaches based on endogenous proteins/peptides. (Created in BioRender. Lietz, S. (2026) https://BioRender.com/77qiisd).

Infection of individuals with the bacteria often occurs through droplets or smears. Upon bacterial infection, the bacteria travel and attach using different virulence factors to the primary site of infection, e.g., the upper respiratory tract and lungs. For successful replication of the bacteria and thus colonization of the host, bacteria produce host-damaging virulence factors, amongst others, so-called exotoxins. Bacterial exotoxins can be classified into three different types (Type I–III) [3,4]. Type I exotoxins cause signaling dysfunction via receptors on cell surfaces. Type II exotoxins damage host cell membranes. Type III exotoxins act intracellularly and are able to enter target cells and directly modify cell functions. The medically highly relevant AB-type protein toxins are type III exotoxins [3,4]. AB-type toxins are highly potent and the causative agents of the clinical symptoms upon bacterial infection. Structurally, AB-type toxins consist of different functional subunits, including an enzymatically active (A) subunit and a binding/translocation (B) subunit (Figure 1). After binding of the AB-type toxin to specific cell surface receptors via its B-subunit, receptor-mediated endocytosis occurs, which results in the uptake of the toxin into cellular compartments. From there, the A-subunit translocates into the cytosol and modifies its specific substrate, in most cases a protein, with very high potency. The toxin-catalyzed substrate modification triggers cellular reactions, which in turn ultimately lead to the development of the typical clinical symptoms associated with the respective disease [5].

Typically, bacterial infections are treated with antibacterial drugs (“antibiotics”, Figure 1). However, the usage of antibiotics is often limited due to various reasons, including the evasion of the immune system through the development of antibiotic-resistant strains [6]. The therapeutic application of antibiotics, for example, in the case of pertussis, must occur as early as possible to successfully manage the infection and consequently the symptoms [7]. As soon as AB-type toxins are released, they are taken up into target cells, and subsequently, the clinical symptoms are caused by the enzymatic activity of the toxin. Since antibiotics are ineffective towards AB-type toxins themselves, they must be applied before the release of the toxin takes place. Anyhow, the early treatment using antibiotics requires early diagnosis and the identification of the pathogen. Regardless, early diagnosis is rare, and therefore, usually, at the start of the antibiotic treatment, the symptoms are already caused. Nonetheless, the application of antibiotics is essential to mitigate or prevent the spread of bacteria within the population.

Further treatment options for toxin-mediated disease are limited and comprise antibodies, which either derived from previous vaccination or due to the application of an antitoxin (e.g., in case of diphtheria antibodies produced in horses). For pertussis treatment, the monoclonal antibodies under evaluation in mice and baboon studies are Hu1B7 and Hu11E6. More detailed information, as well as the advantages and disadvantages of these antibodies, were described elsewhere [8]. However, the antibodies can only bind and thereby neutralize toxin molecules outside cells, since antibodies cannot enter cells and are therefore not able to catch already internalized AB-type toxins (Figure 1). Further clinical interventions are limited to supportive or intensive care to manage disease symptoms. For example, in severe pertussis cases in infants, limited success was reported for extra-corporeal membrane oxygenation or oscillatory ventilation [9,10]. As increasing case numbers are reported and treatment options are limited, toxin-associated diseases are still a matter of concern.

Consequently, novel therapeutic options for the treatment of toxin-mediated diseases are urgently required. These novel therapies could comprise the body’s own protein/peptide-based inhibitors, as the human body might already has developed strategies as part of the innate immune system to combat toxin-mediated diseases. Previously, numerous body’s own proteins and peptides that neutralize clinically relevant bacterial AB-type toxins, including pertussis toxin, diphtheria toxin, anthrax toxin, and clostridial enterotoxins, were identified [11]. This review highlights human proteins and peptides that might be exploited for the future treatment of pertussis.

## 2. The Uptake and Mode of Action of Pertussis Toxin Produced by *Bordetella pertussis*

**Figure 2:**
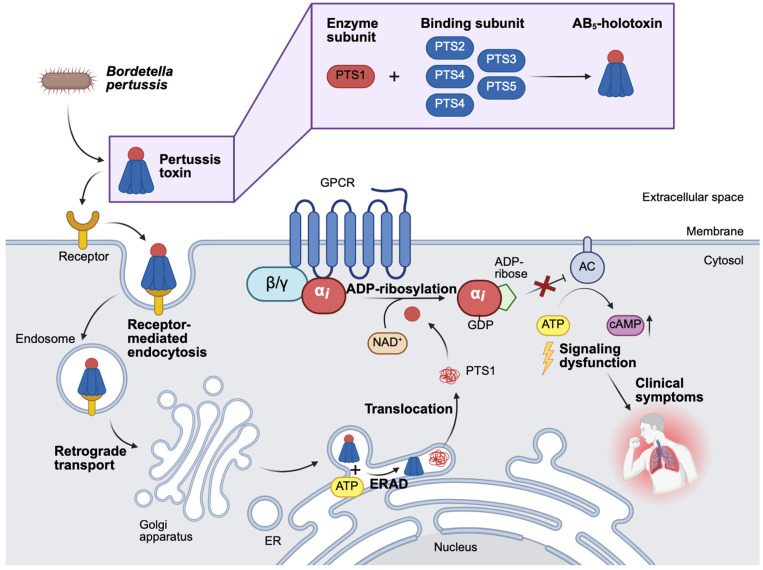
The structure, uptake, and mode of action of PT from *Bordetella pertussis*. *Bordetella* (*B.*) *pertussis* produces, amongst other virulence factors, the AB-type protein toxin pertussis toxin (PT). The AB_5_-holotoxin has one enzyme subunit (PTS1) and five binding subunits S2, S3, S4, and S5 (PTS2–5) which assemble in a 1:1:1:2:1 stoichiometry [14]. Uptake of PT into endosomes occurs after the binding of PT to sialic acid on cell surfaces and receptor-mediated endocytosis. After the retrograde transport of PT to the endoplasmic reticulum (ER), it gets disassembled. Then, PTS1 is transported by the ER-associated degradation pathway (ERAD) into the cytosol, where PTS1 ADP-ribosylates its substrate, inhibitory G-proteins (Gαi) of G-protein-coupled receptors (GPCR), while NAD^+^ serves as a co-substrate. Consequently, Gαi is no longer able to downregulate the adenylate cyclase (AC) activity, causing the enhanced production of intracellular cAMP. Finally, the downstream signaling cascade is altered, and the typical clinical symptoms develop. (adenosine triphosphate (ATP), adenosine diphosphate (ADP), cyclic adenosine monophosphate (AMP) (cAMP), guanosine diphosphate (GDP)) [11,14,30,31,32,33]. (Created in BioRender. Lietz, S. (2026) https://BioRender.com/3th6gu2).

*Bordetella* (*B.*) *pertussis* is a Gram-negative, strictly human pathogen causing the respiratory disease pertussis, also known as whooping cough. Thereby, *B. pertussis* produces several virulence factors that are essential for the development of pertussis disease. One of the most important virulence factors of *B. pertussis* and essential for disease pathology and associated with severe pertussis is the ADP-ribosyltransferase (ART) pertussis toxin (PT) [7,12,13]. As an AB-type protein toxin, PT can be more specifically subclassified due to its structure as AB_5_-holotoxin. PT consists of the enzymatically active A-subunit PTS1 and a binding subunit, the B-pentamer PTS2–4 (Figure 2). In more detail, the B-pentamer is built by five subunits, PTS2, PTS3, PTS4, and PTS5, while PTS4 is present twice. Consequently, the stoichiometry of the PT-holotoxin with its subunits S1:S2:S3:S4:S5 is 1:1:1:2:1 [14,15,16].

After the binding of PT to sialic acid conjugates on cell surfaces, receptor-mediated endocytosis and uptake of PT into endosomes are enabled [17,18]. Subsequently, the holotoxin travels in a retrograde manner from the endosome to the Golgi apparatus and the ER [19,20]. Due to the physiological ATP stores of the ER, ATP attaches to the central pore of the B-subunit, which leads to the disassembly of PT and the release of PTS1. Once released, PTS1, which is thermally unstable, shifts into a disordered/unfolded state, which is recognized by the ER-associated degradation pathway (ERAD), mediating the translocation of PTS1 from the ER into the cytosol [21,22]. The translocation process of PTS1 into the cytosol is assisted by host cell chaperones [23,24,25,26]. Within the cytosol, PTS1 ADP-ribosylates the α-subunit of inhibitory G-proteins (Gαi) associated with G-protein-coupled receptors (GPCRs) using its co-substrate NAD^+^ [27]. Modified Gαi causes the inhibition of the Gαi-mediated inhibition of the adenylate cyclase (AC). Therefore, the AC is activated, and the production of cyclic AMP (cAMP) is enhanced [28]. Elevated cAMP levels are cell type dependent and influence two downstream signaling cascades, protein kinase A (PKA) and the exchange proteins directly activated by cAMP (EPAC) [8,29]. PKA and EPAC signaling are involved in diverse immune and metabolic pathways, causing systemic immunosuppression, lymphocytosis, and metabolic disruptions [29]. Therefore, the downstream signal transduction is altered, and specific symptoms associated with the pertussis disease occur.

## 3. The Relevance of Exploiting Human Endogenous Proteins and Peptides as Part of the Innate Immunity as Novel Inhibitors Against Pertussis Toxin

As previously introduced, despite the vaccination for pertussis, increasing case numbers were reported. The treatment of pertussis in the clinics is solely based on the application of antibiotics and symptomatic therapies, e.g., treating secondary complications of pertussis. Due to the adaptation of pathogens in general, but also *B. pertussis*, increasing antibiotic resistances occur. In the clinics, for the treatment of pertussis, antibiotics belonging to the group of macrolides, such as erythromycin, but also newer macrolides, e.g., azithromycin and clarithromycin, are used [7]. However, there have been reports of macrolide-resistant *B. pertussis* strains. Especially in certain parts of China, between 70 and 100% of clinical isolates showed a macrolide-resistance due to a specific mutation, a 2047 A-to-G mutation within the 23S rRNA gene [34,35,36]. In contrast, outside of China, there have been only sporadic reports on the appearance of macrolide-resistant *B. pertussis* strains [34,36]. Moreover, it is likely that those strains are additionally resistant towards other macrolide antibiotics [7] and that, in the future, further cases of macrolide-resistant *B. pertussis* strains will be reported.

In addition to antibiotic-resistant *B. pertussis*, there were reports of *B. pertussis* escape mutants lacking the virulence factor pertactin. Some acellular pertussis vaccines contain pertactin antigens, which, in addition, contribute to waning immunity after vaccination [6,37,38,39,40].

In general, vaccination is an essential strategy to prevent pertussis. However, infants (<6 months) are a population group at high risk for severe pertussis, since they are unimmunized or only partially immunized [2]. Here, the importance of maternal immunization vaccination programs for pertussis should be communicated early during pregnancy, to protect newborns from pertussis with maternal antibodies [2]. At the same time, for population groups at high risk, effective treatment strategies are urgently required.

Consequently, novel targeted strategies that address the treatment gap that has opened due to evolved *B. pertussis* strains and to protect vulnerable groups at high risk for severe pertussis are urgently required. These strategies are essential for the future treatment of toxin-mediated diseases such as pertussis in the clinics. Because AB-type toxins are the causative agents of the symptoms of the disease, their specific inactivation prevents the clinical manifestation of the disease. Therefore, novel strategies should be specifically directed towards bacterial AB-type protein toxins.

Novel strategies to exclusively target AB-type toxins could comprise endogenous proteins and peptides. Endogenous proteins and peptides are the first line of defense and belong to innate immunity. This involves, for example, antimicrobial peptides that target a diverse set of microbes, such as bacteria or viruses. Since the human body has evolved protein-/peptide-based first line defense strategies to combat, e.g., bacteria, it is likely that the body has also developed strategies against bacterial virulence factors, including AB-type protein toxins. Especially, in regard to the tremendous complexity of the human proteome/peptidome, the odds of identifying the body’s own peptides with anti-toxin activity are extremely high. Due to alternative promoters, splicing, post-translational modifications, and proteolytic processing, the human proteome/peptidome contains more than a million bioactive compounds and, as such, contains huge potential for the discovery of novel protein- and peptide-based therapeutics [41]. Moreover, endogenous proteins and peptides have several benefits regarding their human application, including lower immunogenicity, better safety profile, higher serum stability, better distribution within the human body, and lower production costs [42].

For the identification of novel endogenous human protein/peptide-based inhibitors for PT, two different approaches were exploited. Here, an explorative hypothesis-driven approach and a targeted screening approach were used. Through the explorative hypothesis-driven approach, the group of human α-defensins, specifically α-defensin-1 and -5, was identified as peptide inhibitors of PT [8,43,44]. Moreover, the screening of a human-derived hemofiltrate protein/peptide library allowed the identification of a human protease inhibitor of the SERPIN family, α_1_-antitrypsin (α_1_AT), as a potent inhibitor of PT [31].

## 4. Examples of Human Endogenous Proteins/Peptides and Their Derivatives with Anti-PT Activity

**Figure 3:**
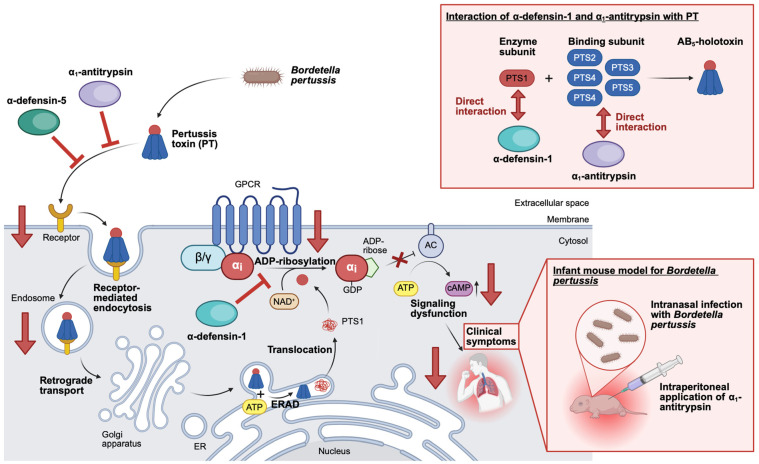
Mode of action of novel protein- and peptide-based inhibitors on PT. α_1_AT and α-defensin-1 and -5 were identified as potent inhibitors for PT. Here, α_1_AT and α-defensin-5 inhibit the binding of PT to cells and, therefore, inhibit subsequent steps of the intoxication cascade. In more detail, α_1_AT prevents the binding of PT to target cells via the direct interaction with the B-pentamer. Moreover, α_1_AT supplementation in pertussis-infected mice led to a reduction in leukocytosis, the hallmark of pertussis disease. In addition, α-defensin-1 inhibits the enzyme activity of PTS1 in vitro. (Created in BioRender. Lietz, S. (2026) https://BioRender.com/0ff7ele).

In the following, examples of human endogenous proteins and peptides or thereof derived peptide derivatives are given, which possess anti-PT activity (Table 1). Subsequently, the mode of action of the single proteins and peptides towards PT is described in more detail (Table 1 and Figure 3). Here, the special focus lies on human α-defensin-1 and -5, as well as human α_1_AT [11].

**Table 1:** Examples for human endogenous proteins and peptides with anti-toxin activity. Human-derived proteins and peptides that inhibit bacterial toxins are listed with the respective mode of action [11].

Human α- and β-Defensins (def)				
Inhibitor	Physiological Function	Rationale Behind Experimental Testing	Mechanism of Inhibition	References
α-def-1, α-def-2, α-def-3, α-def-4, α-def-5	Antimicrobial peptides of the innate immune system are produced by different cell types and organs, depending on the defensin [45,46]	Hypothesis-driven—inhibition of multiple bacterial AB-type protein toxins was previously reported	Inhibition of enzyme activity and inhibition of toxin binding to cells Toxin inhibition, mechanism not tested yet Toxin inhibition, mechanism not tested yet Toxin inhibition, mechanism not tested yet Inhibition of enzyme activity and inhibition of toxin binding to cells	[43,44]
α-def-6, β-def-1, β-def-2			No inhibition No inhibition No inhibition	[43,44]
**Human α_1_-antitrypsin (α_1_AT)**				
**Inhibitor**	**Physiological function**	**Rationale behind experimental testing**	**Mechanism of inhibition**	**References**
α_1_AT	Serin protease inhibitor of the SERPIN-family, activity regulation of serin proteases, e.g., neutrophil elastase within the lungs [47]	Screening	Inhibition of binding	[31]
Antithrombin, antithrombin with fondaparinux	Protease inhibitor of the SERPIN-family, Antithrombin, cleaves thrombin, and fondaparinux acts as an activator of the physiological function of antithrombin, a natural anticoagulant [47,48,49]	Hypothesis-driven—Belongs to the same group of protease inhibitors as α_1_AT	No inhibition	[31]
α_1_AT-HF, α_1_AT-HF P8, 42, 64, (α_1_AT-derived peptides)	No physiological function is attributed	Hypothesis-driven screening	Inhibition of intoxication, due to inhibition of binding (α_1_AT-HF) or a mechanism not yet tested	[50]
VIRIP, and other α_1_AT-derived peptides	VIRIP was previously identified as an inhibitor of HIV [51]		No inhibition	[50]

### 4.1. Group of Defensins as Inhibitors for Pertussis Toxin

Earlier, the scientific community has shown that the group of human defensins has activity in vitro and in vivo against multiple AB-type protein toxins, including, e.g., iota toxin, *Clostridioides* (*C.*) *difficile* toxin (CDT), Toxin A (TcdA) and Toxin B (TcdB), diphtheria toxin (DT), and anthrax lethal toxin (LT) [52,53,54,55,56,57,58]. Here, the mechanism of action of the different defensins towards the bacterial toxins was often related to the formation of aggregates and binding of the bacterial toxin, as well as the inhibition of the enzyme activity of the toxin through the defensins. Therefore, testing whether the defensins have activity against PT was a hypothesis-driven approach, since a broad set of bacterial toxins had already been inhibited by the group of defensins.

Human endogenous defensins are antimicrobial peptides (AMPs) that are involved as part of the innate immune system in the first line defense in a non-specific way against a broad spectrum of pathogens, including bacteria, fungi, and viruses [45]. In more detail, human defensins showed antibacterial activity against a variety of Gram-negative and Gram-positive bacteria. Here, most often the mechanism of action of the human defensins towards the bacteria is related to the disruption of bacterial membranes or the inhibition of cell wall synthesis [46]. During these processes, the human defensins target structures such as the outer membrane proteins, LPS, peptidoglycan, lipid II, phosphatidylglycerol, and lipoteichoic acids, as well as bacterial DNA and RNA [46]. To reach their target, namely the pathogens in the human body, defensins need to be produced, stored, transported, and released when needed at specific pathogen entry sites. Human defensins are located alongside other antimicrobial factors within the azurophilic granules of neutrophils or are secreted by other cell types, e.g., epithelial cells, amongst others of the kidneys, pancreas, and female reproductive tract or by Paneth cells found in the small intestine at the bottom of the crypt [45,46]. A more comprehensive overview of the structure, expression sites of different defensins and the attributed function was summarized earlier by others and would exceed the scope of this review [45,46]. Briefly, the human defensins can be grouped according to their cysteine pairing and their structure into α-, β-, and θ-defensins [59,60]. They are small (16–50 amino acids), arginine-rich, cationic peptides with a β-sheet structure and three intramolecular disulfide bridges [45,59,60]. For the following, relevant defensins are the α-defensins (3–4 kDa, 29–35 amino acids) and the β-defensins (3–5 kDa, 38–50 amino acids) [45]. The α-defensins are further divided into α-defensin-1-4, also termed Human Neutrophil Peptides (HNPs) and the α-defensin-5 and 6, also termed Human (enteric) Defensins (HDs) [46], while the β-defensins are also known as human β-defensins (HBDs).

In our hypothesis-driven approach, selected α- and β-defensins were tested for their anti-PT activity by analyzing the ADP-ribosylation status of Gαi using CHO-K1 or A549 cells. When investigating the effect of α- and β-defensins towards PT in this assay, it was shown that α-defensin-1, -2, -3, -4, and -5 have inhibitory potential against PT, but not α-defensin-6 and β-defensin-1 and -2 [43,44]. Subsequently, α-defensin-1 and -5 were selected as lead candidates for further mechanistic evaluation, while the results of Kling and colleagues are briefly summarized in the following [8,43,44]. When α-defensin-1 and -5 were preincubated with PT before addition to cells, the inhibitory effect on ADP-ribosylation of Gαi was even stronger. Moreover, the inhibition of ADP-ribosylation of Gαi mediated by α-defensin-1 and -5 occurred in a concentration-dependent manner. An alternative endpoint to determine the PT intoxication of cells is the measurement of PT-mediated effects on cAMP signaling in the living cell-based interference in the Gαi-mediated signal transduction (iGIST) assay. The results of the iGIST assay further confirmed that α-defensin-1 and -5 are inhibitors of PT, since the two peptides caused a decrease in cAMP signaling in the presence of PT. Moreover, α-defensin-1 and -5 but not β-defensin-1 inhibited the enzyme activity of PTS1 in vitro using recombinant Gαi in a concentration-dependent manner. However, the inhibition of enzyme activity mediated by α-defensin-1 was stronger than the inhibition mediated by α-defensin-5. Most interestingly, the inhibition of ADP-ribosylation of Gαi in PT-treated living cells and of enzyme activity of PTS1 in vitro mediated by α-defensin-1 and -5 was lost when linearized versions of α-defensin-1 and -5 were used. The linearized versions of α-defensin-1 and -5 had no disulfide bonds as the cysteine residues were replaced with serine residues, which indicates that the three-dimensional structure of the defensins is important for mediating the inhibition of PT. Furthermore, the effect of α-defensin-1 and -5 on PT binding to cells was investigated using flow cytometry and fluorescence microscopy. Here, both defensins showed inhibition of binding and uptake of PT into cells, while the inhibitory effect mediated by α-defensin-5 was stronger compared to the effect mediated by α-defensin-1. In addition, it was shown that α-defensin-1 is taken up into different cell lines in a stronger manner than α-defensin-5. Intracellularly, the signal for α-defensin-1 and PTS1 but not α-defensin-5 and PTS1 are in proximity, as shown by a proximity ligation assay. The close proximity of α-defensin-1 and PTS1, but not of α-defensin-5 and PTS1, was further supported by in silico modeling, demonstrating that α-defensin-1 but not α-defensin-5 contains specific interaction interfaces with PTS1. Those results were additionally confirmed by in vitro interaction analysis using dot plot experiments. Collectively, the results showed that α-defensin-1 is able to enter cells, reach and interact with its target PTS1.

Consequently, the inhibitory effect of α-defensin-1 on PT was mainly mediated via inhibition of the enzyme activity of PTS1, while α-defensin-5 mainly inhibited cellular uptake of PT. Therefore, the inhibitory mode of action for α-defensin-1 and -5 slightly differs but complements each other. Since no adverse effects on cell viability by α-defensin-1 and -5 were detected, both human endogenous defensins are promising candidates for further evaluation regarding the treatment of pertussis in the clinics.

### 4.2. α_1_AT and Derived Peptides as Inhibitors for Pertussis Toxin

Through the screening of a human hemofiltrate peptide library and the application of bioassay-guided fractionation, as well as mass spectrometry analysis, α_1_AT was identified as an inhibitor of PT [31].

As mentioned above, α_1_AT (52 kDa) is a highly abundant human serin protease inhibitor of the SERPIN superfamily, which is mainly produced by hepatocytes of the liver, but also other cell types [61,62,63]. The physiological function of α_1_AT is to bind and therefore inhibit serine proteases, including neutrophil elastase, proteinase 3, cathepsin G, trypsin, and transmembrane serine protease 2 (TMPRSS2) [47,64,65,66]. For example, the serine protease neutrophil elastase targets components of the extracellular matrix, e.g., elastin and collagen [67]. The inhibition of neutrophil elastase by α_1_AT leads to the protection of cells and tissues from proteolytic activity and degradation, e.g., within the lungs [68]. Consequently, α_1_AT is a major regulator of protease activity and modulator of inflammatory responses in the human body, protecting the lungs from neutrophil elastase released by immune cells [69]. Therefore, the acute phase protein α_1_AT combats and prevents inflammation-induced tissue damage, while physiological plasma concentrations of ~17–38 μM can increase 4–5-fold upon acute inflammation [47,70]. Individuals suffering from the genetic disorder α_1_AT deficiency have lower serum levels of α_1_AT, which results in unregulated tissue degradation in the lower respiratory tract [71]. The α_1_AT deficiency can be treated by the intravenous augmentation of α_1_AT, which is obtained and purified from donor blood, using licensed drugs, e.g., Prolastin^®^. For such drugs, administration protocols and safety profiles are already well-established [72].

After the identification of α_1_AT through an unbiased screening approach, the inhibitory mode of action of α_1_AT towards PT was investigated in detail. This experimental validation involved different in silico, in vitro, and in vivo methods, while corresponding results are briefly summarized [31]. For these experiments, the approved drug Prolastin^®^ was used as a source for α_1_AT, while being referred to as α_1_AT in the following. First, the effect of α_1_AT as an isolated compound was investigated in a cell-based assay. This assay analyzed the ADP-ribosylation status of Gαi, the specific substrate of PT, in PT-treated CHO-K1 cells and the more physiologically relevant lung adenoma A549 cells in FCS-free conditions. Here, a concentration-dependent inhibition of ADP-ribosylation of Gαi in PT-treated cells was observed for both cell lines. This effect was even stronger when α_1_AT was preincubated with PT before the addition to cells. Moreover, the inhibitory effect of α_1_AT towards PT was preserved when repeating the assay in FCS-containing conditions. Notably, α_1_AT had no adverse effects on the cell viability of CHO-K1 cells after 4 and 24 h and no effect on the detection of ADP-ribosylated Gαi. To analyze whether the protective effect of α_1_AT was related to its protease activity, another member of the SERPIN superfamily, antithrombin, was tested for its ability to inhibit PT. Anyhow, the protease activity of α_1_AT was most likely not mediating the inhibition of PT, as neither antithrombin alone nor the combination of antithrombin with its activator fondaparinux inhibited the ADP-ribosylation of Gαi in PT-treated CHO-K1 cells. Moreover, α_1_AT showed only minor influences on the enzyme activity of PTS1 in vitro, independent of whether recombinantly purified Gαi or CHO-K1 cell lysate was used as a source for Gαi. Subsequently, the uptake of PT into target cells was investigated in more detail, using flow cytometry and fluorescence microscopy. In those experiments, it was shown that α_1_AT inhibits the binding of PT to CHO-K1 and A549 cells in a concentration-dependent manner. Here, the preincubation of α_1_AT with PT before addition to cells increased the inhibition of PT-binding to CHO-K1 cells even more, indicating that the interaction of PT and α_1_AT during the preincubation might be relevant. Using high-resolution stimulated emission depletion (STED) microscopy, it was demonstrated that signals for PT and α_1_AT partially colocalize, particularly visible when higher α_1_AT concentrations were used. To investigate whether there is an interaction between α_1_AT and PT in vitro and in silico, interaction analysis was conducted. The dot blot analysis, where α_1_AT was vacuum-aspirated onto a membrane and overlayed with PT or PTS1, revealed that α_1_AT exclusively interacts with the PT holotoxin but not with its isolated enzyme subunit, PTS1. This suggests that the interaction of PT and α_1_AT is most likely mediated by the B-subunit of PT. This result is further strengthened by the in silico analysis, where a blind docking analysis was performed during which α_1_AT was allowed to explore the surface of PT. This analysis suggests as well that the B-pentameric ring of PT, especially subunit 3 (PTS3), is important for the α_1_AT-PT interaction. Finally, an infant mouse model for pertussis was employed to investigate the effect of α_1_AT in vivo. Upon infection of infant C57BL/6 mice with *B. pertussis*, the mice develop pertussis-associated leukocytosis, the hallmark of pertussis disease, which is also observed in human infants with pertussis. Using this animal model, expression levels for the murine α_1_AT genes (serpinA1a-e) were downregulated in *B. pertussis*-infected infant mice in direct comparison to non-infected mice. Furthermore, when *B. pertussis*-infected infant mice were treated with α_1_AT, the white blood cell count, used to assess leukocytosis, was reduced in comparison to infected vehicle-treated mice.

Consequently, α_1_AT inhibits intoxication of target cells by inhibiting the uptake of PT through direct interaction with PT. Thus, α_1_AT prevents subsequent steps of the intoxication cascade and ultimately reduces the development of pertussis-associated symptoms in an in vivo mouse model for pertussis. Further studies will examine α_1_AT levels in humans who have developed pertussis disease and whether augmentation of reduced or low α_1_AT levels might benefit the disease outcome.

A further study revealed that α_1_AT inhibits further PT-related bacterial AB-type protein toxins, including C2 toxin from *C. botulinum*, DT from *C. diphtheriae*, and a *B. anthracis* fusion toxin [73]. This further highlights the potential of α_1_AT as a pan-toxin inhibitor and the prospect of α_1_AT-containing drugs for the treatment of pertussis and other toxin-mediated diseases (diphtheria and anthrax) in repurposing approaches.

Finally, the amino acid region of full-length human endogenous α_1_AT that bears anti-PT activity was identified [50]. For that purpose, α_1_AT-derived peptides spanning different parts of the full-length α_1_AT were generated and tested for their ability to inhibit PT by analyzing the ADP-ribosylation status of Gαi in PT-treated CHO-K1 cells. Through this approach, three α_1_AT-derived peptides termed α_1_AT-HF, 42, and 64 were identified to inhibit the ADP-ribosylation of Gαi in PT-treated CHO-K1 cells. Another previously published peptide derived from α_1_AT, termed VIRIP [51] showed no inhibition of PT. The amino acid region of α_1_AT that bears anti-PT activity was confirmed by an overlap of a total of nine peptides of various lengths. The lead peptide α_1_AT-HF is endogenously present in human hemofiltrate and has the shortest sequence of α_1_AT with anti-PT activity. Interestingly, this peptide was shown to have no adverse effects on cell viability and in vivo in zebrafish embryos, rendering it a perfect candidate for further modification and optimization approaches for human application.

The findings with PT provided further proof-of-concept that the generation of peptides based on endogenous human protein precursors can result in the generation of peptides with anti-toxin activity, which has a scientific and clinical impact for various protein toxins from other medically highly relevant bacteria, e.g., *C. difficile*, which are also neutralized by human peptides and proteins [73,74].

## 5. Conclusions

This comprehensive review summarizes the recent efforts to identify and optimize the human body’s own peptides and proteins as novel inhibitors of PT, the highly potent and specific virulence factor of *B. pertussis*, responsible for the severe (childhood) disease pertussis. These results from basic research revealed novel aspects of human innate immunity, as the identification of human endogenous proteins and peptides as natural inhibitors of bacterial toxins underlines and highlights the role of the innate immune system to fight not only bacteria but also their produced virulence factors. Moreover, the novel findings should be transferred for the development of novel pharmacological strategies against pertussis. Such novel therapeutic approaches are urgently required as the current approaches, including vaccination, antibiotic treatments, and symptomatic treatment strategies, are losing their effectiveness, causing an increase in reported pertussis cases worldwide.

## Data Availability

No new data were created or analyzed in this study.

## Abbreviations

α_1_AT	α_1_-antitrypsin
Gαi	α-subunit of inhibitory G-proteins
ART	ADP-ribosyltransferase
ADP	Adenosine diphosphate
AMP	Adenosine monophosphate
ATP	Adenosine triphosphate
AC	Adenylate cyclase
AMPs	Antimicrobial peptides
*B. anthracis*	*Bacillus anthracis*
*B. pertussis*	*Bordetella pertussis*
CHO-K1 cells	Chinese hamster ovary cells strain K1
*C. difficile*	*Clostridioides difficile*
CDT	*Clostridioides difficile* toxin
cAMP	Cyclic AMP
def	Defensin
DT	Diphtheria toxin
ER	Endoplasmic reticulum
ERAD	ER-associated degradation
ECDC	European Center for Disease Prevention and Control
EPAC	Exchange proteins directly activated by cAMP
GDP	Guanosine diphosphate
GPCRs	G-protein-coupled receptors
HBDs	Human β-defensins
HDs	Human (enteric) Defensins
HNPs	Human Neutrophil Peptides
iGIST	Interference in Gαi-mediated Signal Transduction
LT	Lethal toxin
PT	Pertussis toxin
PTS1	Pertussis toxin enzyme subunit
PTS2–5	Pertussis toxin binding subunits S2, S3, S4, and S5
PKA	Protein kinase A
TcdA	Toxin A
TcdB	Toxin B
